# Congenital Transmesenteric Hernia With Closed-Loop Small Bowel Obstruction and Intestinal Gangrene in an Adult

**DOI:** 10.7759/cureus.53560

**Published:** 2024-02-04

**Authors:** Siddharth V Songadkar, Dency S Hansalia, Haryax V Pathak, Jitesh A Desai

**Affiliations:** 1 General Surgery, Shree Krishna Hospital, Pramukhswami Medical College, Bhaikaka University, Karamsad, IND

**Keywords:** internal hernia, management of transmesenteric internal hernia, transmesenteric hernia, intestinal gangrene, small bowel obstruction, congenital transmesenteric hernia

## Abstract

Internal hernias (IHs) are defined as the protrusion of the intestine through an aperture in the peritoneal ligament, mesentery, or the omentum, without traversing the fascial planes and leading to encapsulation of the protruded intestine within another compartment of an otherwise intact abdominal cavity. Internal hernias can be acquired or congenital. Congenital internal hernias resulting in small bowel obstruction are extremely rare, even more so in adults, occurring usually due to embryological or developmental defects, often undiagnosed, and requiring a high index of clinical suspicion. We report a rare case of a 22-year-old young adult with transmesenteric hernia of distal ileum through a congenital distal ileal mesenteric defect resulting in closed-loop small bowel obstruction and distal ileal gangrene, managed with resection and end-ileostomy, followed by stoma reversal one month later. It requires a high index of suspicion and remains a surgical emergency due to its predilection for intestinal gangrene and small bowel obstruction, with a high mortality rate due to delayed presentation and diagnosis.

## Introduction

Internal hernia (IH) is defined as the protrusion of the gut through an aperture in the peritoneal ligament, mesentery, or the omentum, without traversing the fascial planes and leading to encapsulation of the protruded gut within another compartment of an otherwise intact abdominal cavity. IH can be acquired or congenital. The incidence of IH is less than 1%, including both congenital and acquired IHs, and they are responsible for approximately 0.6-5.8% of all cases of small bowel obstruction (SBO) [1].

Congenital IHs are rarer, even more so in adults, accounting for barely 8% of all iHs, usually due to embryological or developmental defects, often undiagnosed, and require a high index of clinical suspicion [2]. Congenital IHs are a common cause of SBO in infants and pediatric age groups, but are extremely rare among adults, with only 28 cases documented in published literature [3-17]. They often remain undiagnosed, usually being an intra-operative diagnosis, and hence require a low threshold for surgical intervention. Late diagnosis is associated with a higher mortality rate, nearly 50%, due to resulting SBO and bowel ischemia [18].

We report a case of a 22-year-old male presenting with complaints of severe acute abdominal pain and signs of acute intestinal obstruction, later diagnosed as closed-loop SBO, and intestinal gangrene due to a congenital transmesenteric hernia.

## Case presentation

A 22-year-old male was brought into the emergency department of a local primary care private hospital with sudden onset, generalized, abdominal pain for three days, associated with abdominal distension and multiple episodes of vomiting. The patient was initially managed conservatively for three days and then referred to our hospital for further management in view of the worsening clinical condition of the patient and the lack of advanced facilities at the primary care hospital.

Upon presentation at our hospital, the patient had generalized abdominal pain, non-radiating, associated with multiple episodes (approximately 9-10) of bilious vomiting over the previous two days, and reduced appetite. The patient recounted a history of similar symptoms, on and off over the last 1.5 years, with a history of constipation for one year, but had not availed medical consultation for the same. The patient reported no history of fever, changes in bowel habits, no urinary complaints, and no complaints of hematemesis or per-rectal bleeding. The patient had no other abdominal surgeries, no history of recent or past abdominal trauma, and no significant family history or history of medications.

The patient had abdominal distension, generalized tenderness, guarding, and an ill-defined, tender, palpable, infra-umbilical ovoid lump, approximately 5x3cm in size, which was intra-abdominal, non-reducible, and non-mobile in nature. He exhibited signs of septic shock, and was hemodynamically unstable, with blood pressure of 100/70 mmHg, heart rate of 115 bpm, and a respiratory rate of 22 per minute, maintaining adequate oxygen saturation on room air. His blood investigations showed leucocytosis and metabolic acidosis (Table 1, Table 2).

**Table 1:** Routine hematological investigations suggestive of sepsis

Parameters	Patient values	Reference values	Unit
Total Leucocyte Count	19.3	4-10	x1000/ul
Red Blood Cell Count	4.7	4.5-5.5	million/cmm
Hemoglobin	13.6	13-17	g/dl
Hematocrit	40	36-46	%
Platelet Count	322	150-410	x1000/ul
Neutrophils	84	40-80	%
Lymphocytes	12	20-40	%
Eosinophils	1	2 – 6	%
Monocytes	3	2-8	%
Basophils	0	0-1	%
C-reactive Protein	123	<3	mg/L
Sodium	140	136-145	mmol/L
Potassium	3.2	3.8-5.2	mmol/L
Chloride	102	96-106	mmol/L
Serum creatinine	1.04	0.80-1.30	mg/dl
Prothrombin time	10.48	9.42-12.62	Seconds
International normalized ratio	1.1		
Activated partial thromboplastin time	29.9	22.86-31.42	Seconds

**Table 2:** Arterial blood gas analysis showing metabolic acidosis pCO2: partial pressure of carbon dioxide; pO2: partial pressure of oxyge; Na+: sodium; K+: potassium; HCO3: bicarbonate

Parameters	Patient value	Reference values	Unit
pH	7.28	7.38-7.44	
pCO2	38	35-45	mmHg
pO2	98	80-100	mmHg
Na+	140	136-145	mmol/L
K+	3.2	3.8-5.2	mmol/L
HCO3	18	22-28	mEq/L
Lactate	1.4		mmol/L

**Figure 1:**
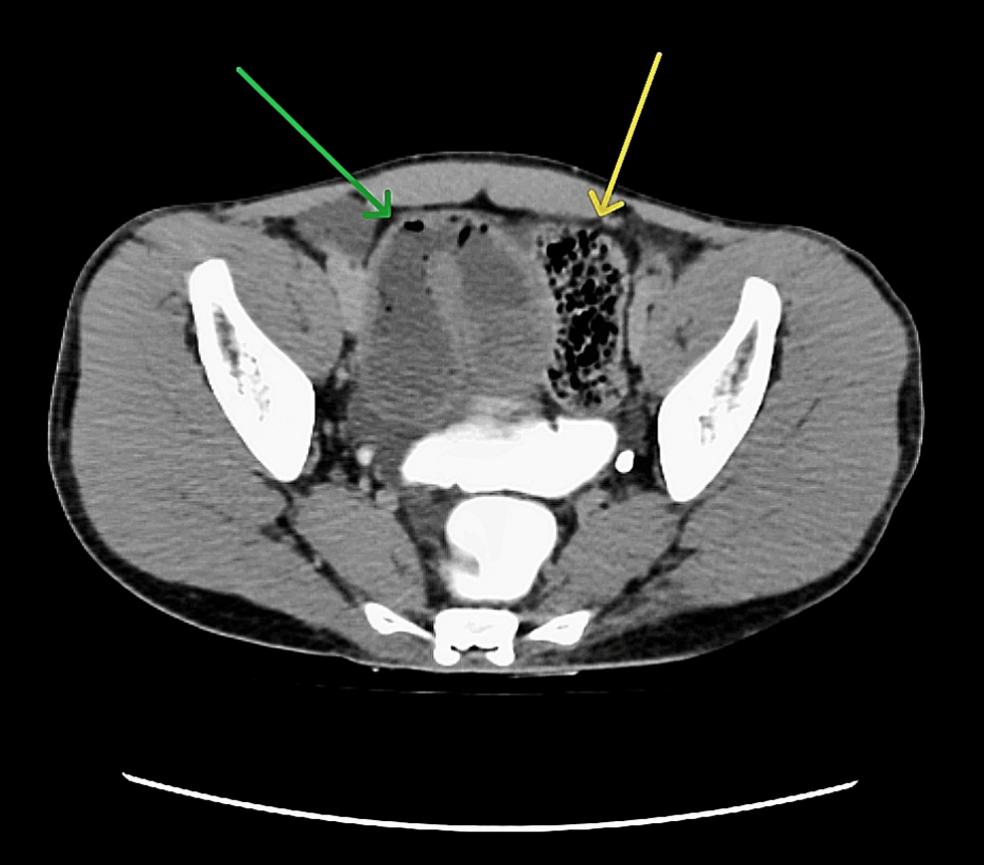
CECT scan of abdomen and pelvis (axial section) CECT shows a closed loop of small bowel in the pelvis (green arrow), with gross dilatation of the involved small bowel loops and thickened, edematous bowel wall. Small bowel feces sign is visible in adjacent bowel loops (yellow arrow), which is the presence of fecal matter intermingled with gas bubbles in the lumen of small bowel, occurring due to reduced intestinal motility and obstruction to the passage of fecal material. These features are suggestive of closed-loop small bowel obstruction. CECT: contrast-enhanced computed tomography

The patient had already undergone a contrast-enhanced computed tomography (CECT) scan of the abdomen during his admission at the previous hospital that suggested SBO (Figure 1).

In view of clinical examinations and the CT findings, the patient was promptly shifted for surgery.

**Figure 2:**
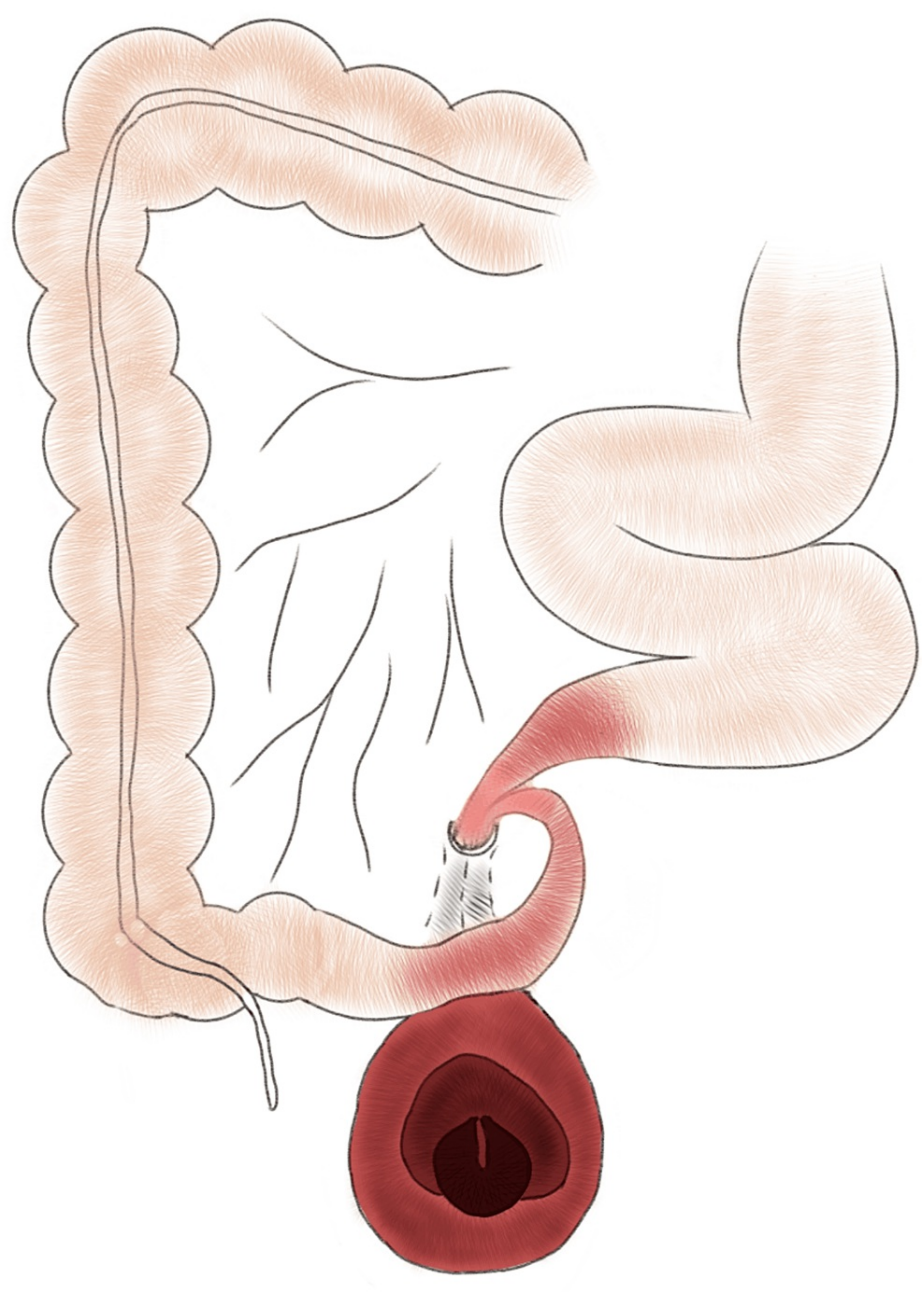
Schematic representation showing the anatomy of the hernia Image credit: Haryax Pathak (author)

**Figure 3:**
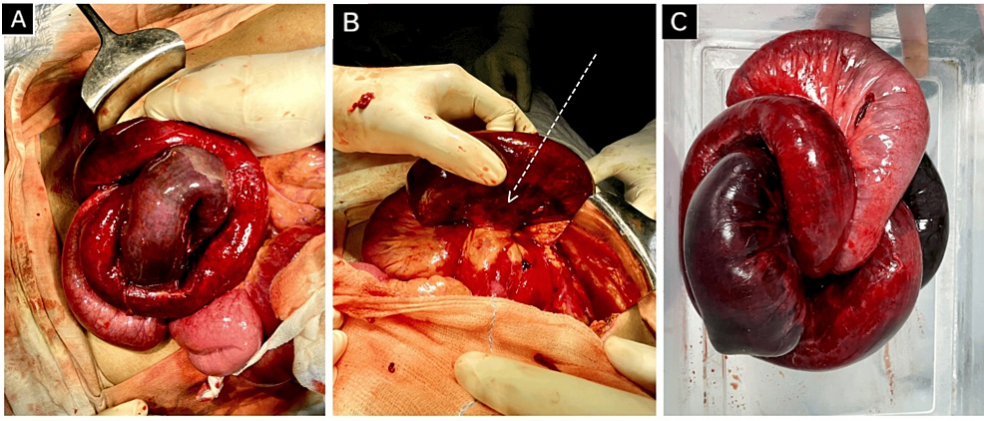
Intra-operative findings (A) Obstructed hernia with signs of gangrene and ischemia; (B) White arrow showing constriction ring at mesenteric defect causing strangulation and ischemia; (C) Resected segment showing "Gordian Knot" of herniated intestine

**Figure 4:**
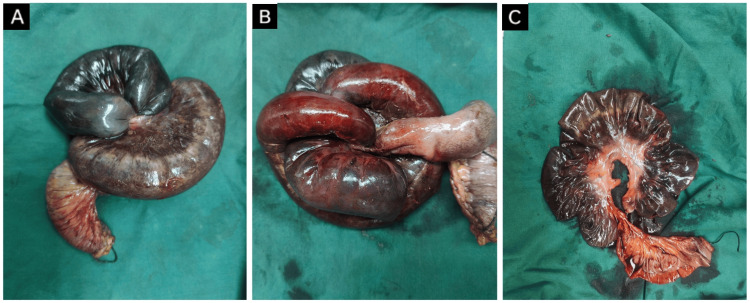
Gross examination of resected segment (A) Superior aspect; (B) Inferior aspect; (C) Post de-rotation image of intestinal loops showing mesenteric defect in the center

Emergency exploratory laparotomy was performed that revealed a gangrenous ileal segment herniating through a congenital defect in the mesentery of the distal ileum, with a constricting ring at the defect that was responsible for strangulation of the herniated segment leading to SBO and ileal gangrene. This herniated segment had twisted upon itself twice, forming a triple helical structure, described in the literature as the “Gordian knot of herniated intestine” [2] (Figure 2, Figure 3, Figure 4). It was approximately 380 cm distal to the duodenojejunal flexure and 5 cm proximal to the ileocecal (IC) junction, measuring approximately 30 cm in length. The peritoneal cavity was inflamed, with the presence of foul-smelling fluid, suggestive of toxic peritonitis.

In view of frank peritonitis, and to prevent ensuing toxemia, reduction of the herniated segment was avoided. As the segment was gangrenous, a decision was made to go for resection of the herniated segment. Considering the patient’s poor general condition and the increased morbidity associated with peritonitis, the distal end of the ileum at the IC junction was closed in two layers and the proximal end of the ileum was brought out in the right iliac fossa as an end ileostomy. Following the surgery, the patient was observed in the ward for five days and discharged; he exhibited good recovery with regular weekly follow-up.

Histopathological examination of the resected segment showed ischemic changes of the small bowel, with no evidence of vasculitis or thrombosis in the mesenteric vessels.

**Figure 5:**
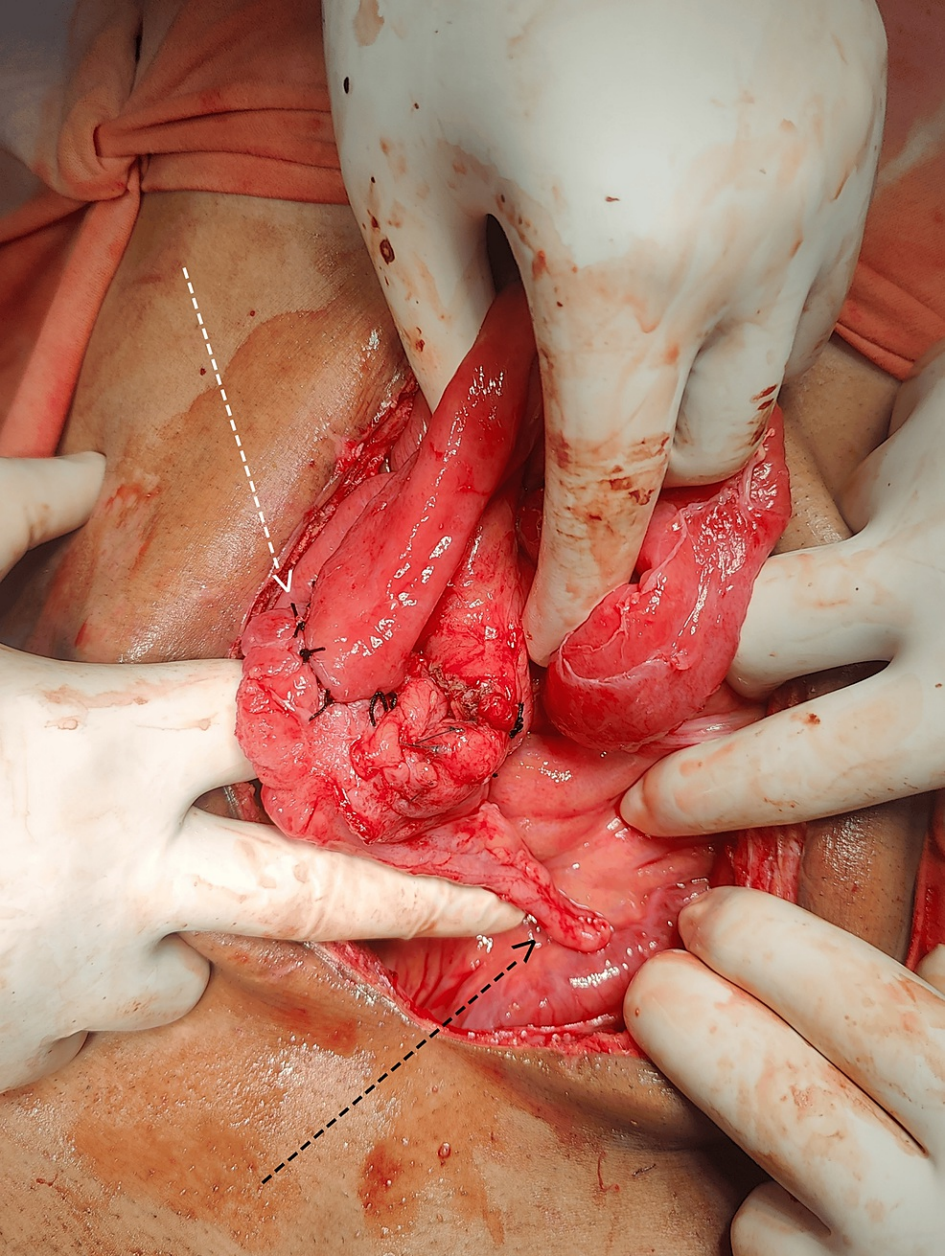
End-to-side Ileocecal anastomosis Stoma reversal was done and end-to-side anastomosis of the end-ileostomy was done with the caecum. White arrow: end-to-side Ileocecal anastomosis; Black arrow: appendix

The patient underwent a successful surgery for stoma reversal one month later with IC anastomosis (Figure 5). Post-operatively the patient made an uneventful recovery. The patient remained in regular follow-up for one month following the surgery and showed signs of complete recovery.

## Discussion

IHs are broadly classified into two categories as described by Ghahremani: (a) Congenital and (b) Acquired, and are further divided into six types: (i) Paraduodenal, (ii) Foramen of Winslow, (iii) Pericecal, (iv) Intersigmoid, (v) Transmesenteric, and (vi) Retroanastomotic. Further studies describe a more detailed and varied classification of internal hernias, especially congenital IHs, dividing them into intraperitoneal and retroperitoneal subtypes, including extremely rare presentations like paravesical, trans-omental, trans-falciform, broad ligament, etc. [19].

The pathogenesis of congenital IH remains unclear. Rokitansky, in 1836 described the first case of a congenital transmesenteric hernia, diagnosed at autopsy, wherein the cecum had herniated through a mesenteric defect near the IC valve. Treves, in 1885, described the Treves Field, an area in the terminal ileal mesentery, circumscribed by the junction of the ileocolic artery and the last branch of the ileal artery, an area devoid of fat, blood vessels, or lymph nodes, and highly susceptible to injury during the developmental phase [20]. Proposed theories speculate vascular insult in the prenatal period that subsequently leads to ischemic changes and thinning of the mesenteric leaves, as prenatal intestinal ischemia is found to be associated with bowel atresia in 5.5% of the pediatric population. A few other hypotheses have been proposed like a regression of dorsal mesentery, mesenteric compression by the colon during the fetal midgut herniation, and rapid lengthening of a particular segment of the mesentery. It may even be associated with other congenital anomalies like cystic fibrosis or Hirschsprung disease [1].

As described earlier, congenital IHs are rare, and usually present in the neonatal or pediatric age group. The first instance of a congenital transmesenteric hernia presenting in an adult was reported by Moulay et al. in 1971 [4]. The incidence of congenital IHs presenting with SBO and bowel ischemia in adulthood is extremely rare, with <30 case reports in published literature up to 2023, to the best of our knowledge [3-17]. Since there are so few case reports documented and published, no systematic reviews outlining the presentation, ideal course of management, or outcomes are available for comparison. Patients present with non-specific symptoms like abdominal pain, bloating, nausea, vomiting, constipation, or even diarrhea in some cases. Clinical examination is usually unremarkable with few patients exhibiting signs of SBO or intestinal gangrene, like abdominal distension, tenderness, and guarding. Few patients may even present with a palpable lump in the lower abdomen, as in this patient, described in the literature as the "Gordian Knot of herniated intestine” [1]. Radiological investigations like contrast radiographs, ultrasonography, and CECT may be helpful in diagnosing SBO and intestinal ischemia. It requires a high index of clinical suspicion to diagnose congenital transmesenteric hernia. The final diagnosis is ultimately made once the patient is taken for surgery. Time is of the essence in such cases as the patient may appear clinically stable, but may deteriorate rapidly, thus leading to mortality.

Once taken for surgery, the surgeon must not forget the basic tenets of general surgery. The usual approach adopted by most surgeons, as evident from the published reports, is to reduce the herniated segment or resect the segment if gangrenous and proceed with primary anastomosis. In this case, the patient had intestinal gangrene and frank peritonitis with hemodynamic instability. The patient was at risk of subsequent toxemia and increased risk of morbidity; hence it was decided to resect the gangrenous segment, close the distal loop, and bring out the proximal end as an end-ileostomy. Therefore, it is imperative to approach such cases based on clinical evaluation and individual characteristics rather than simply following a textbook approach based on the variety of the hernia itself.

As per an analysis by Newsom et al., congenital IHs with SBO and intestinal gangrene are associated with a nearly 50% mortality rate, most often due to delayed presentation and diagnosis [18]. Hence, congenital transmesenteric hernias, especially presenting in adulthood with SBO and intestinal gangrene require accurate clinical examination, rapid investigation, a high index of clinical suspicion, and a low threshold for surgical intervention for the best possible management of the patient and better prognostic results.

## Conclusions

Congenital transmesenteric hernias can present in adults, especially with features of SBO and intestinal gangrene, and must not be ruled out as a diagnosis simply on the basis of the age of presentation. They are associated with a high mortality rate if diagnosed late or left untreated. As pre-operative diagnosis is difficult for patients with no history of previous abdominal trauma or surgeries presenting with SBO, surgeons must exhibit a high index of clinical suspicion for congenital transmesenteric hernias and push for early surgical intervention to allow for better prognostic outcomes.
